# Lectures on the Diagnosis and Treatment of Diseases of the Heart

**Published:** 1841-12-18

**Authors:** W. W. Gerhard


MEDICAL EXAMINER.

DEVOTED TO MEDICINE, SURGERY, AND THE COLLATERAL SCIENCES.

No. 51.] PHILADELPHIA, SATURDAY, DECEMBER 18, 1841. [Vol. IV.

### LECTURES ON THE DIAGNOSIS AND TREATMENT OF DISEASES OF THE HEART.

BY W. W. GERHARD, M. D.

### LECTURE XXI.

The disorders of other organs than the heart nearly all depend upon the anormal condition in which the whole vascular system is placed; and result mainly from the fulness of the capillary system. Thus cephalalgia is a very frequent accident; when it occurs, it is usually contusive, deep seated, and accompanied with giddiness, flushing of the face, and other signs of active congestion. But as soon as the blood has become watery, congestion of the brain scarcely takes place except after exercise, or other causes which excite the circulation. Absolute rest will almost always prevent it. The tendency to congestion of the lungs has been already mentioned as a thoracic symptom: still it is secondary to the disorders of the heart proper, and only indirectly connected with the disease.

The viscera of the abdomen rarely escape. The disorder of the liver is much more frequent than that of any other organ of this cavity; in its simplest form it is a mere engorgement, and even distension of the vessels of the organ from the difficulty in the venous circulation; hence the organ enlarges, is indurated, and of course interferes with the due performance of digestion, and, to a certain extent, impedes respiration. But if the engorgement continue, the usual change in the condition of the liver takes place, its nutrition is deeply altered, the acini in part enlarge, and become indurated, and in part are pressed upon by those which are already increased in size; it loses the deep-red colour of early congestion, and assumes a yellowish tint; in other words, decided cyrrhosis is formed. The symptoms proper to the liver, as jaundice, &c., are often developed, and the case might be mistaken for one of original disease of this organ, unless the attention is drawn to the history of the symptoms and their evident starting point in the heart itself. Flatulence is another frequent symptom; it seems to arise from the disordered digestion which often accompanies hypertrophy, and is more marked than in other affections, because the impediment to the action of the heart, which arises from the distended abdomen, causes extreme dyspnœa. The kidneys frequently participate in the disorder; generally they are not diseased until the liver has previously suffered, and the affection then becomes a triple one, which is almost always followed by dropsical effusion much earlier than the uncomplicated disease of the heart. The disease of the kidneys ends, if it does not begin, by the peculiar alteration of the cortical substance known under the name of granular degeneration, which is singularly analogous to the cyrrhosis of the liver, and seems to depend much upon the same causes.

*Progress and termination.*—In most cases, hypertrophy, if once developed, especially if of the least simple kind, tends to pass to a more advanced stage, and the progress of the disease is, therefore, towards a fatal termination. This is almost always the case, unless the patient remains for a long time at perfect rest; death may then take place in one of the two ways, either suddenly, or more slowly, from gradual exhaustion and dropsical effusions. In the simple varieties, the progress of the disease may be arrested, and, at most, it is so slow that it scarcely shortens the ordinary duration of life, unless it should produce some secondary disorders of other viscera.

*Diagnosis and Prognosis.*—This evidently depends mainly on the physical signs; for, although the oppressive palpitations and disagreeable sensations of the patient, are all more or less indicative of the disorder; none of these signs are sufficient; even when taken together, they are rather uncertain. The physical signs depending on measurement of extent, and of impulsion, are much more easy and certain, and can rarely lead into error. The chances of mistake are, in fact, reduced almost to nothing, if the physician examines the patient at several times, and finds that the signs vary but little. For the strong impulsion of a hypertrophied heart is very different from the quick jerking motion of one that is merely excited by some temporary functional disturbance. The prognosis of hypertrophy is so completely dependent upon the progress of the disorder as to require very few remarks. If the patient retain a full vascular system, without suffering from extreme dyspnœa on moderate exercise, and without a constant tendency to visceral engorgements, the prognosis is least unfavourable. If he become anemic, and still suffer from much palpitation and oppression, the prognosis becomes extremely grave, for the strength declines while the local disease continues unabated. The rapidity of increase of the heart, and the ossification of the vessels also increase the danger, especially if the latter occur in the valves of the heart, as well as in the arteries.

*Treatment.*—Hypertrophy being either originally a disease of nutrition, or becoming so after a previous inflammatory disorder, it is very clear that no active antiphlogistic treatment can be decidedly curative. Indeed all treatment is simply moderative, designed to check the stimulation of the heart, and allow the natural powers to recover themselves, and, if possible, to regain the balance which is lost.

The principal indication is, therefore, to keep the heart as quiet as possible, by withdrawing all unnecessary stimulants, and by moderating that excess of action and of nutritive life which it has already acquired. Many of our most important means are, therefore, strictly hygienic, and these are amongst those whose utility is unquestionable.

A patient who is affected with hypertrophy, must be informed that no sudden cure is possible, and that the amelioration of his symptoms must depend in a great measure upon the energy of his will, and his perseverance in watching the influence of stimulating agents upon the heart. He should carefully avoid all active violent exercise, all extreme mental agitation, the use of stimulating drink, or of an exciting highly animalized diet, coffee, tobacco and other nervous excitants, amongst which excessive venereal indulgence is one of the most powerful. Moderate exercise by walking, driving, or riding at an easy pace, is not to be forbidden, except for a short period during some temporary excitement of the heart, and the diet should not be extremely rigid ; it must be plain, but sufficient, with animal food in moderate quantity once daily.

In moderately severe cases, the hygienic treatment, with the occasional application of cups to the region of the heart, will best ensure the comfort of the patient. Cups may be used whenever the action of the heart becomes oppressed, or its impulsion decidedly increased. General blood-letting will produce relief under the same circumstances, and is habitually resorted to by many patients ; but there are some decided objections to its employment. If prescribed frequently for the relief of slight cardiac disturbance, it must be again resorted to whenever the necessity returns ; and it will be required more and more often as the patient becomes more feeble, and the blood less rich ; for it is a general law, that venesection will relieve a chronic disease of the heart by lessening the quantity of blood which obstructs the action of the organ, but it increases its irritability, and enfeebles the patient. It is, therefore, really a necessary evil. The objections to the use of cups are by no means so strong, nor does the application of them produce the same necessity of frequent repetition.

In all cases in which the disease is not positively active, we restrict our remedies to those just mentioned, but from time to time the hypertrophy seems to advance rapidly, the patient suffers more, and the throbbing is almost incessant. This results from accidental inflammation, or from temporary excitement. Bloodletting is then sometimes necessary to relieve the oppression, but it must be regarded as a purely temporary remedy, not to be repeated unnecessarily, or carried to a large amount. After a moderate bleeding to diminish the violent throbbing of the heart and arteries, the patient should keep as quiet as possible during the attack, not taking active exercise of any kind. By these means the temporary excitement may in general be speedily removed.

The use of digitalis is at times indicated, but, like bloodletting, it is better adapted for the removal of temporary attacks of palpitation and excitement, than for the radical cure of the disorder. If it be administered for a long period, it either loses, in a great degree, its peculiar control over the heart, or it must be urged to an extent which is dangerous, from the uncertainty of its action when accumulated in the economy. For these reasons I have in a great measure abandoned the digitalis as a permanent remedy, restricting it to a few cases in which it acts decidedly, in small doses; and even in these cases I prefer giving it with assafœtida or camphor, than to prescribing it alone.

But when the hypertrophied heart is excited, either from inflammation or other causes, digitalis is safe and highly useful as an adjuvant to more certain antiphlogistic means. It may be prescribed in the usual doses of ten drops of the tincture four times a day, or two-thirds of a grain at the same intervals. I have never in these doses seen any inconvenience result from it where its action was watched. The infusion as usually prepared is extremely uncertain, but if it be made carefully, and of given strength, with the addition of a sufficient quantity of sugar to make it into a syrup, it is preferable to the tincture. After the temporary excitement of the heart is quieted, the digitalis may be laid aside for a time, and again resumed when a like necessity arises.

The object, then, of the treatment, is to remove the causes which increase the tendency to hypertrophy; to keep the heart as quiet as possible, and to arrest the causes which hasten the progress of the disease by giving a new activity to the growth of the heart. The clothing of the patient must be carefully attended to; without being oppressively warm, it should obviate as much as possible the rheumatic attacks which favour heart diseases.

